# Occurrence and Distribution of Three Low Molecular Weight PAHs in Caño La Malaria, Cucharillas Marsh (Cataño, Puerto Rico): Spatial and Seasonal Variability, Sources, and Ecological Risk

**DOI:** 10.3390/toxics13100860

**Published:** 2025-10-11

**Authors:** Pedro J. Berríos-Rolón, Francisco Márquez, María C. Cotto

**Affiliations:** Nanomaterials Research Group, Department of Physics, Chemistry, and Mathematics, School of Natural Sciences and Technology, Universidad Ana G. Méndez-Gurabo Campus, Gurabo, PR 00778, USA; berriosp1@uagm.edu

**Keywords:** polycyclic aromatic hydrocarbons, urban wetland, surface water, seasonal variability, ecological risk assessment

## Abstract

Polycyclic aromatic hydrocarbons (PAHs) are persistent organic pollutants with significant ecological and public health implications, particularly in urban wetlands exposed to chronic anthropogenic stress. This study evaluates the occurrence, spatial distribution, seasonal variability, and ecological risk of three low molecular weight PAHs—naphthalene (NAP), phenanthrene (PHEN), and anthracene (ANT)—in surface waters of Caño La Malaria, the main freshwater source of Cucharillas Marsh, Puerto Rico’s largest urban wetland. Surface water samples were collected at four locations during both wet- and dry-season campaigns. Samples were extracted and quantified by GC-MS. NAP was the dominant compound, Σ3PAHs concentrations ranging from 7.4 to 2198.8 ng/L, with higher wet-season levels (mean = 745.79 ng/L) than dry-season levels (mean = 186.71 ng/L); most wet-season samples fell within the mild-to-moderate contamination category. Compositional shifts indicated increased levels of PHEN and ANT during the wet season. No significant spatial differences were found (*p* = 0.753), and high correlations between sites (r = 0.96) suggest uniform input sources. Diagnostic ratios, inter-species correlations, and principal component analysis (PCA) consistently indicated a predominant pyrogenic origin, with robust PHEN–ANT correlation (r = 0.824) confirming shared combustion-related sources. PCA revealed a clear separation between dry- and wet-season samples, with the latter showing greater variability and stronger associations with NAP and ANT. Ecological risk assessment using hazard quotients (HQ_water_) indicated negligible acute toxicity risk across all sites and seasons (<0.01); the highest HQ_water_ (0.0095), observed upstream during the wet season, remained within this range. However, benchmark exceedances by PHEN and ANT suggest potential chronic risks not captured by the acute ERA framework. These findings support integrated watershed management practices to mitigate PAH pollution and strengthen long-term ecological health in tropical urban wetlands.

## 1. Introduction

Polycyclic aromatic hydrocarbons (PAHs) are semi-volatile organic compounds with two or more fused aromatic rings, broadly classified into low molecular weight (LMW, 2–3 rings) and high molecular weight (HMW, ≥4 rings), a distinction that governs their environmental behavior [1]. LMW-PAHs, due to their higher solubility and mobility, are generally more abundant in surface waters, whereas HMW-PAHs are particle-associated, prone to sediment accumulation, and often occur near or below detection limits [2,3]. They originate from natural and anthropogenic sources, mainly incomplete combustion of fossil fuels and biomass (pyrogenic), petroleum refining and spills (petrogenic), and, to a lesser extent, biogenic synthesis by microorganisms and plants [4,5]. PAHs are widely dispersed across atmospheric, terrestrial, and aquatic systems [6,7,8]. Transport processes such as atmospheric deposition, urban runoff, and wastewater discharge drive their accumulation in sediments and subsequent transfer through food webs [9,10,11]. Due to their persistence, hydrophobicity, and toxic properties, PAHs are classified as priority pollutants by the United States Environmental Protection Agency (USEPA) and are of global concern for their mutagenic, teratogenic, and carcinogenic effects [12,13]. These risks are especially critical in shallow urban wetlands, where stormwater inflows and limited dilution capacity exacerbate ecological exposure [14,15,16].

Urban wetlands, located within or near densely populated areas, function as transitional ecosystems that provide critical services such as water filtration, flood control, and habitat for aquatic organisms [17]. Because of their proximity to multiple anthropogenic emission sources, they are highly vulnerable to contamination by PAHs, receiving inputs from traffic emissions, industrial discharges, and unregulated stormwater runoff [14,18]. In this context, they act both as reservoirs and conduits for pollutants, while also serving as natural ecological filters that can decrease PAHs through microbial degradation, sedimentation, and plant uptake under favorable conditions [19].

Elevated concentrations of PAHs have been reported in urban rivers, lakes, and drainage canals across Asia, Africa, and Latin America, frequently surpassing international water quality standards [20,21,22]. Enclosed or semi-enclosed wetlands—such as urban marshes—often retain higher PAH loads due to restricted hydrological exchange, shallow waters, and organic-rich sediments, which enhance pollutant retention, sedimentary accumulation, and long-term ecological exposure [23,24]. However, in tropical urban wetlands—where environmental dynamics are intensified by climatic variability and human pressures—integrated assessments remain scarce, with most existing studies focused on sediments and biota [25], rather than dissolved LMW-PAHs in surface waters.

For example, coastal sediments in Trinidad showed higher concentrations during the wet season [26], while Venezuelan mangrove soils at Tucacas Bay exhibited moderate-to-high PAH levels, likely enhanced by freshwater inflows [27]. Other subtropical mangrove wetlands in Brazil reported moderate sediment contamination linked to navigation and domestic effluents, with hydrodynamic conditions shaping spatial distribution [28], whereas river sediments in Brazilian urban catchments reflected strong vehicular inputs, particularly naphthalene (NAP) dominance [29]. In Cuba, Santana et al. [30] documented LMW-PAH dominance in Almendares River surface waters, with polluted sites exceeding acute ecological risk thresholds, in contrast to the Gulf of Batabanó where Tolosa et al. [31] found low sediment levels dominated by natural organic matter sources. The present study addresses this gap by providing the first spatial and seasonal assessment of dissolved LMW-PAHs in surface waters of Puerto Rico’s largest urban wetland.

Spatial and seasonal assessments of LMW-PAHs such as NAP, phenanthrene (PHEN), and anthracene (ANT) are still limited in tropical urban wetlands [32,33]. Due to their phase behavior and environmental mobility, these compounds are readily transported during rainfall events, which facilitates their dispersion and often results in higher detection frequencies in the dissolved phase compared to sediments, where they may occur at lower concentrations due to their greater solubility and lower particle affinity [34,35]. In tropical urban environments, high temperatures and intense land-use pressures may amplify their ecological risks, through seasonal microbial degradation dynamics and transient contamination pulses [36,37,38,39]. However, studies that also quantify the ecological risk associated with LMW-PAH concentrations in tropical island wetlands remain notably absent.

Caño La Malaria is a tidal-influenced urban waterway within the highly urbanized Cucharillas Marsh watershed in Cataño, Puerto Rico, which plays a critical role in connecting upland runoff with the San Juan Bay Estuary [40,41,42]. It receives runoff from roads, informal settlements, and industrial zones before discharging into the San Juan Bay Estuary. Prior studies have reported elevated levels of fecal coliforms, heavy metals, and untreated sewage [42,43,44], but PAHs have never been characterized in its surface waters. Given its location within a protected natural reserve, Caño La Malaria offers a relevant case study for evaluating the spatial and seasonal variability of these compounds in an ecologically vulnerable wetland system.

Although PAHs are widely recognized as significant aquatic pollutants, field assessments in tropical island environments remain scarce. Particularly in Puerto Rico, existing research has focused primarily on estuarine sediments and amphibian habitats [45,46,47,48], with no studies addressing the occurrence or seasonal variability of PAHs in waters of urban channels like Caño La Malaria, despite previous recommendations [49] and recent evidence of human exposure [50,51]. This urban waterway represents a critical yet understudied conduit of pollutant transfer into a protected tropical urban wetland. Within this context, this study addresses a critical knowledge gap by evaluating the occurrence, spatial distribution, seasonal trends, and potential sources of three LMW-PAHs—NAP, PHEN, and ANT—in Caño La Malaria. These compounds were prioritized due to their higher solubility and relevance as indicators of dissolved-phase contamination, while acknowledging that regulatory frameworks such as the USEPA’s 16 priority PAHs also include HMW congeners more relevant to sediments and biota [52,53,54]. In addition, it integrates an ecological risk assessment (ERA) based on measured surface water concentrations and toxicity thresholds to estimate potential impacts on aquatic organisms. These findings support future monitoring and restoration efforts in tropical urban wetlands under similar anthropogenic pressures. This need is further emphasized by the lack of region-specific studies and long-term environmental assessments of PAH contamination in Caribbean urban wetlands, underscoring the urgency of sustained monitoring and management efforts [55].

## 2. Materials and Methods

### 2.1. Study Area and Sampling Design

Caño La Malaria is a tidal-influenced estuarine channel located in the municipality of Cataño, on the northern coast of Puerto Rico (18.4450° N, 66.1178° W). It functions as the primary hydrological conduit for surface water flow from the Cucharillas Marsh watershed into the San Juan Bay Estuary [42]. The channel receives year-round surface water inputs from upland tributaries such as the Santa Catalina and San Diego streams [56], which contribute to a continuous outflow into San Juan Bay. Its hydrological regime is artificially regulated by tide gates and a pump system located at the outlet, which limit marine–terrestrial exchange, block tidal inflow, and promote freshwater retention under seasonal rainfall conditions [40,41]. The region follows a bimodal precipitation pattern characteristic of Puerto Rico and the wider Caribbean, with a dry season (December–March), early rainfall (April–July), a brief midsummer drought, and late rainfall season (August–November) [57,58].

**Figure 1:**
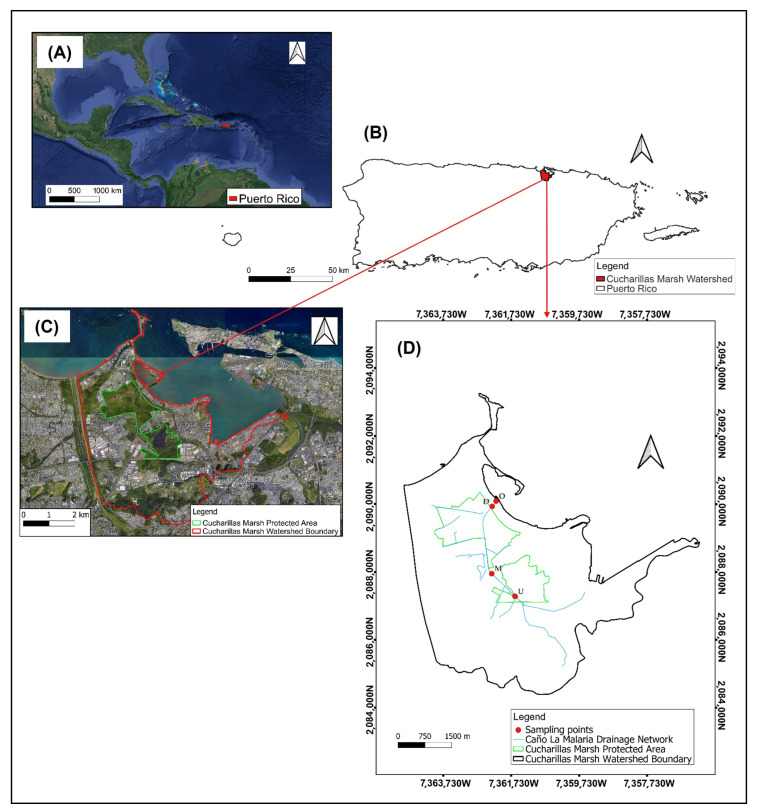
Geographic location and spatial context of the study area within the Cucharillas Marsh watershed, Cataño, Puerto Rico. Panel (**A**) shows the location of Puerto Rico within the Caribbean region. Panel (**B**) highlights the position of the Cucharillas Marsh watershed on the island of Puerto Rico. Panel (**C**) presents the watershed boundary (red) and protected wetland area (green) over satellite imagery. Panel (**D**) displays the drainage network of Caño La Malaria (blue), the Cucharillas Marsh Protected Area boundary (green), and the Cucharillas Marsh watershed boundary (black). Red dots indicate surface water sampling stations corresponding to U, M, D, and O segments along the Caño La Malaria system. Maps prepared using publicly available data from GIS Puerto Rico [59].

Surface water samples were collected at four fixed sampling sites—upstream (U), midstream (M), downstream (D), and outlet (O)—representing spatial variation from upstream headwaters to the channel’s outlet. Site selection was based on proximity to stormwater discharge points, urban infrastructure, and accessibility during both dry and wet seasons. A total of four sampling campaigns were conducted in March, September, and November 2022, and February 2023, corresponding to two dry- and wet-season events. At each sampling station and campaign, duplicate surface water samples were collected. Figure 1 illustrates the geographic context of the study area, including watershed boundaries, drainage features, and the spatial distribution of surface water sampling sites along Caño La Malaria.

### 2.2. Materials and Reagents

Surface water samples were collected in pre-assembled amber wide-mouth glass bottles (LabForce^®^, Thomas Scientific, Swedesboro, NJ 08085, USA), equipped with PTFE-lined caps to prevent analyte adsorption and minimize photodegradation of PAHs during transport and storage. All laboratory glassware used in sample collection, extraction, and concentration was thoroughly cleaned following a multi-stage protocol. This included sequential washing with 1% (*w*/*v*) Alconox^®^ detergent solution (White Plains, NY 10603, USA), rinsing with distilled water, repeated rinses with deionized water, acid rinsing with 50% nitric acid to remove metal and organic residues, followed by additional deionized water rinses, and a final rinse with acetone. The glassware was subsequently dried in a laboratory oven at 100 °C for a minimum of 2 h prior to use. The liquid–liquid extraction procedure employed certified HPLC-grade dichloromethane (DCM; CAS No. 75-09-2; GFS Chemicals, Powell, OH 43065, USA). Anhydrous sodium sulfate (CAS No. 7757-82-6; Thermo Fisher Scientific, Waltham, MA 02452, USA), was used to remove residual water from the organic extracts. Analytical standards of PAHs, including NAP, PHEN, and ANT, were obtained as a certified solution at 1000 µg/mL in DCM (Agilent Technologies, Santa Clara, CA 95051, USA, Method 610-compliant), and were used for calibration and quality control of gas chromatography-mass spectrometry (GC-MS) quantification procedures.

### 2.3. Sample Preparation and Extraction

PAHs were extracted from 1-L surface water samples within 48 h of collection to minimize degradation, using a liquid–liquid extraction adapted from the USEPA standard method [52]. Each sample was transferred to a 2000 mL separatory funnel and extracted with three portions of 60 mL of DCM. The combined organic phases were dried with anhydrous sodium sulfate, added incrementally until no residual water remained, as indicated by freely moving granules, followed by gravity filtration through Whatman #1 filter paper. The dried extract was concentrated to dryness using a rotary evaporator equipped with a water bath maintained at 30–35 °C. The residue was then reconstituted in 5 mL of DCM and transferred to amber glass vials. Extracts were stored at 4–6 °C in a laboratory-grade refrigerator and analyzed within 7 days. Each extract was injected in triplicate into the GC–MS.

### 2.4. GC-MS Instrumental Analysis

LMW-PAHs were quantified by GC–MS using a Shimadzu GC-2010 Plus–QP2020 system (Shimadzu, Kyoto, Japan). Separation of the target compounds was achieved using a Restek Rxi-5Sil MS capillary column (30 m × 0.25 mm i.d., 0.25 µm film thickness; Restek Corporation, Bellefonte, PA, USA) with high-purity helium (99.999%) as the carrier gas at a constant linear velocity of 43.7 cm/s. The injection was conducted in splitless mode with an injector temperature of 300 °C, and an injection volume of 1 µL was delivered using an AOC-20i autosampler (Shimadzu, Kyoto, Japan). The oven temperature program was set as follows: initial temperature of 90 °C (held for 2 min), ramped at 5 °C/min to 320 °C, with a final hold time of 12 min, for a total runtime of 60 min. The MS was operated in electron ionization (EI) mode at 70 eV, with an ion source temperature of 230 °C and an interface temperature of 300 °C. The solvent cut time was set at 3.5 min. Data acquisition was performed in Selected Ion Monitoring (SIM) mode, targeting characteristic *m*/*z* values of 128 (NAP), 178 (PHEN and ANT). Isomeric differentiation between PHEN and ANT was achieved by comparing retention times to those of certified standards.

### 2.5. Data Processing and Statistical Analysis

All GC-MS chromatograms were processed using the Postrun Analysis program of GC-MS solution software, version 2.6 (Shimadzu Corporation, Kyoto, Japan), to quantify the peak areas and retention times of the target PAHs. External calibration curves were constructed using certified PAH standards (NAP, PHEN, and ANT) at known concentrations in DCM. The coefficient of determination (R^2^) for each compound exceeded 0.99. Quantified concentrations were compared against national and international water quality guidelines to assess the ecological significance. The Shapiro–Wilk test indicated that ∑3PAHs concentrations were not normally distributed (W = 0.76, *p* < 0.01, *n* = 16), supporting the use of non-parametric statistical tests. Accordingly, the Friedman test was applied to assess spatial and seasonal differences in PAH concentrations, using a significance level of α = 0.05. All statistical analyses were performed using Microsoft Excel (Microsoft 365; Microsoft Corporation, Redmond, WA, USA).

### 2.6. Quality Assurance and Quality Control

External calibration curves for each PAH were prepared daily using at least seven concentration levels (0.10–100.0 ng/L), achieving high linearity (R^2^ > 0.99). The limit of detection (LOD) and limit of quantification (LOQ) were determined for each analyte and batch using the method based on the standard deviation of the intercept divided by the slope of the calibration curve [60]. LOD and LOQ were calculated using the formulas: LOD = 3.3 × SE/m and LOQ = 10 × SE/m, where SE is the standard error of the y-intercept and m is the slope of the calibration curve. All reported concentrations above LOQ were considered quantifiable. LOD values ranged from 0.02 to 0.59 ng/L, and LOQ values ranged from 0.06 to 1.80 ng/L across the four sampling campaigns (see Supplementary Table S2).

Blanks consisting of HPLC-grade DCM were injected into GC-MS throughout each run to confirm the absence of carryover and instrument background contamination. No target PAHs were detected in the blanks. Analytical precision was assessed through duplicate sample acquisition and triplicate injections in the GC-MS. Relative standard deviation (RSD) and coefficient of variation values were calculated for each sample (see Supplementary Table S3). Nondetected (ND) values in individual injections were treated as zero for mean concentration calculations.

### 2.7. Ecological Risk Assessment

To estimate the potential ecological risk associated with NAP in surface waters of Caño La Malaria, we applied the Hazard Quotient (HQ) method [61,62,63,64]. The HQ was calculated as follows:

*HQ_water_* = *EC_water_*/*PNEC_water_* (1)

*PNEC_water_* = *L(E)C*_50_/*AF* (2)

EC_water_ represents the maximum concentration of NAP detected in surface water (ng/L). The predicted no-effect concentration in water (PNEC_water_) was derived from acute toxicity data for *Callinectes sapidus* (blue crab), a native estuarine decapod crustacean widely distributed across Puerto Rico’s coastal wetlands [65]. Based on the 96-h LC_50_ (lethal concentration for 50% of test organisms), the lowest observed L(E)C_50_ was 0.68 mg/L, applying an assessment factor (AF) of 10, and yielding a PNEC_water_ of 0.068 mg/L (68,000 ng/L) [66]. Toxicity data for NAP, were obtained from the ECOTOX database [67].

HQ_water_ values were then calculated for each site and sampling date using the maximum observed NAP concentrations to reflect worst-case exposure scenarios. Ecological risk categories were assigned following the classification criteria proposed by Li et al. [64]. The categories used are summarized in Table 1.

## 3. Results

### 3.1. Spatial and Seasonal Variability in PAH Concentrations

Table 2 presents the concentrations of NAP, PHEN, and ANT across four seasonal sampling campaigns and four stations along Caño La Malaria. PAH concentrations exhibited marked variability, with higher levels recorded during the wet season (September and November 2022) compared to the dry season (March 2022 and February 2023). For instance, Σ3PAHs during the dry season ranged from 7.40 ng/L (U, March) to 363.11 ng/L (O, February), while wet season values spiked up to 929.57 ng/L (M, September) and peaked at 2198.83 ng/L (U, November), driven by extreme ANT concentrations (up to 1313.60 ng/L). NAP was the most abundant compound, reaching 485.10 ng/L (U, September), although its dominance shifted to ANT in November. PHEN varied broadly (ND–557.43 ng/L), with notable peaks at M and U sites. The O consistently registered Σ3PAHs between 43.43–712.54 ng/L across all campaigns, underscoring its role as the final receptor before discharge into San Juan Bay.

**Figure 2:**
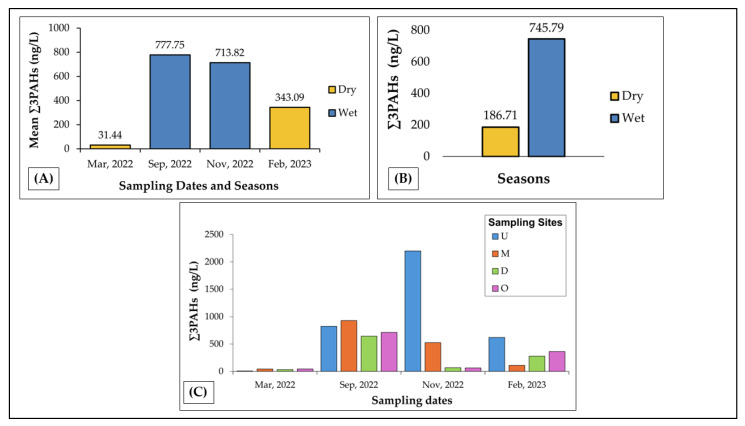
Seasonal and spatial variation of total LMW-PAHs (Σ3PAHs: NAP, PHEN, and ANT) in surface water samples collected from Caño La Malaria. (**A**) Mean Σ3PAHs concentrations (ng/L) per sampling campaign. (**B**) Comparison of overall mean Σ3PAHs concentrations by season. Overall means were calculated as the arithmetic average of all Σ3PAHs values measured across the four sampling sites within each season (*n* = 8 values per season). (**C**) Spatial and seasonal distribution of Σ3PAHs across four sampling sites (U, M, D, O) and four campaigns.

Figure 2 presents the seasonal and spatial distribution of Σ3PAHs concentrations in surface waters of Caño La Malaria. Panel A illustrates that mean Σ3PAHs concentrations were elevated during the wet season, reaching 777.75 ng/L in September and 713.82 ng/L in November, in contrast to lower values observed in the dry season—31.44 ng/L in March and 343.09 ng/L in February. Panel B further underscores this seasonal pattern, showing that overall mean Σ3PAHs concentrations during the wet season (745.79 ng/L) were more than threefold higher than those recorded during the dry season (186.71 ng/L). Panel C depicts the spatial variation across the four sampling sites, with site U consistently exhibiting the highest concentrations, particularly in November 2022, whereas sites D and O showed persistently lower levels throughout the sampling period.

A Friedman test confirmed seasonal differences in Σ3PAHs concentrations across the four sampling campaigns (χ^2^ = 21.00, *p* = 4.59 × 10^−6^), supporting the trend of elevated PAH levels during the wet season. In contrast, no significant differences were found among the sampling sites (χ^2^ = 1.20, *p* = 0.753), suggesting a homogeneous spatial distribution of PAHs across Caño La Malaria, despite marked seasonal variability.

### 3.2. PAH Sources and Compositional Patterns

#### 3.2.1. Compositional Patterns

**Figure 3:**
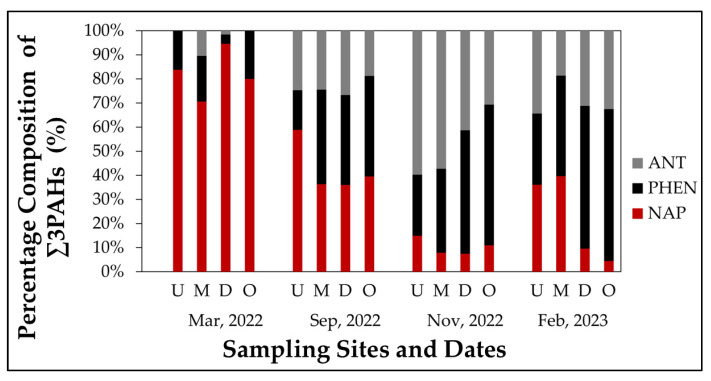
Percentage composition of individual LMW-PAHs—NAP, PHEN, and ANT—in surface water samples collected from Caño La Malaria. Data is shown for four sites: U, M, D, O, across four sampling campaigns. Each bar represents the relative contribution (%) of each PAH to the total Σ3PAHs concentration.

Figure 3 illustrates the relative percentage composition of the three LMW-PAHs across all sampling sites and dates. During the first sampling campaign (March 2022), NAP dominated the ∑3PAHs composition at all sites, representing 80–100% of the total concentration. PHEN contributed at sites M and O (16–20%), while ANT was either absent or accounted for less than 10%. By September 2022, the compositional profile became more balanced, especially at sites U and M, where NAP accounted for approximately 59% and 36%. PHEN and ANT showed increased contributions at these sites during the wet season (e.g., PHEN: 16–39%; ANT: 25–24%).

In November 2022, ANT became the dominant compound at all sites, notably at U and M, where it represented approximately 60% and 57% of ∑3PAHs. PHEN also remained elevated (25–58%), while NAP accounted for less than ~20% of the total. In February 2023, PHEN was the most abundant PAH at sites D and O, contributing approximately 59% and 63% of ∑3PAHs, followed by ANT with 31% and 32%. NAP contributions were highly variable: low in November and February (6–15%), but much higher in March (70–98%) and September (28–59%). Across all campaigns, NAP represented between 4% and 98% of Σ3PAHs, dominating during the March 2022 campaign. PHEN and ANT showed greater seasonal and spatial variability, with ANT being notably higher during the peak wet season and PHEN becoming more dominant at downstream sites in the final sampling campaign.

#### 3.2.2. Sources by Diagnostic Ratios

**Figure 4:**
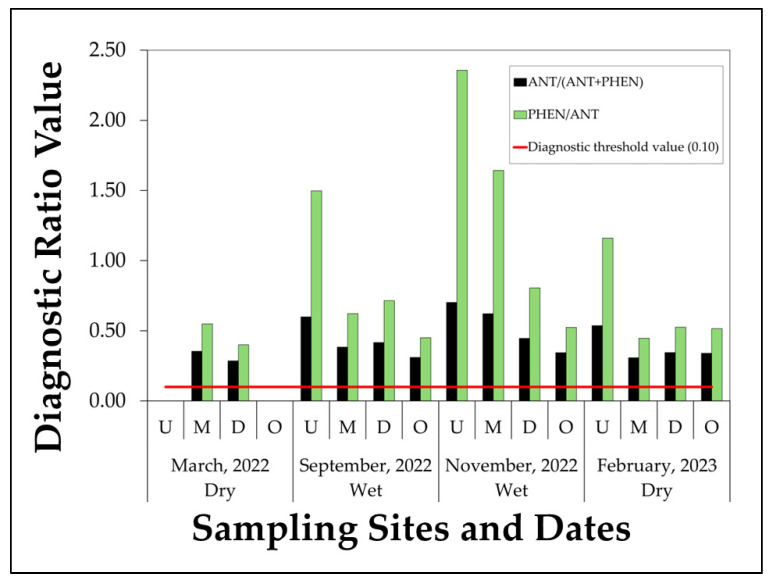
Diagnostic ratios of PAHs used for emission source attribution in surface water samples from Caño La Malaria, collected at four sites—U, M, D, O—during the seasonal campaigns (March, September, November 2022, and February 2023). Bars represent the value generated by the diagnostic ratios used: ANT/(ANT + PHEN) (black) and PHEN/ANT (green). The red line indicates the threshold value of 0.10 for ANT/(ANT + PHEN), above which pyrogenic sources are inferred. PHEN/ANT values below 10 also suggest a pyrogenic origin.

PAH source apportionment was evaluated using two diagnostic ratios: ANT/(ANT + PHEN) and PHEN/ANT. The former distinguishes petrogenic sources (<0.10) from pyrogenic ones (>0.10), while the latter indicates pyrogenic inputs when values are below 10. As illustrated in Figure 4, all ANT/(ANT + PHEN) values exceeded the 0.10 threshold, indicating a consistent pyrogenic signature across Caño La Malaria’s surface waters. While PHEN/ANT ratios were also below 10, with most values under 2.50. Elevated values in both ratios aligned with periods of higher Σ3PAHs concentrations. In March 2022, anthracene was not detected at sites U and O (Table 2), which prevented the calculation of diagnostic ratios requiring both PHEN and ANT. For this reason, ratios are only interpreted for sites and campaigns where both compounds were quantifiable.

#### 3.2.3. Sources by Principal Component Analysis

**Figure 5:**
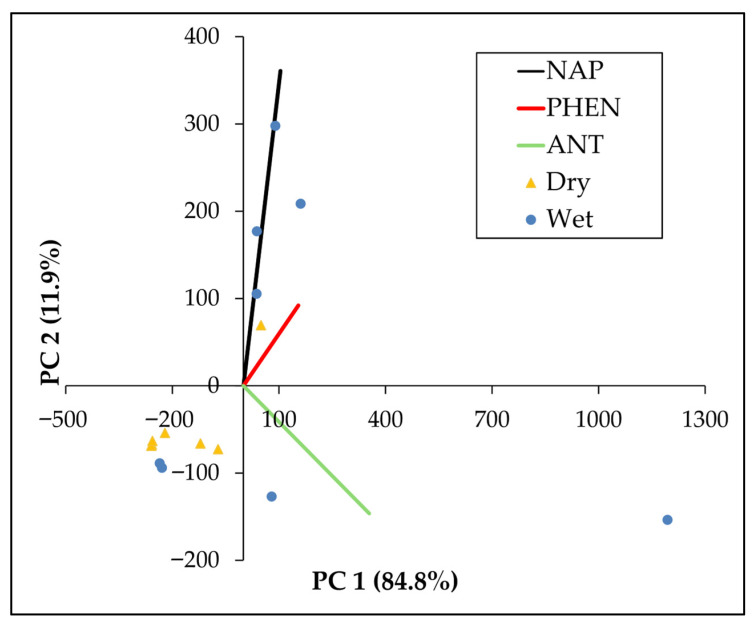
PCA biplot of surface water samples from Caño La Malaria based on concentrations of three LMW-PAHs: NAP, PHEN, and ANT. The plot displays the distribution of 16 samples grouped by season (Dry vs. Wet) along the first two principal components (PC 1 and PC 2), which explain 84.8% and 11.9% of the total variance, respectively. Vectors represent the directional contribution of each PAH to the multivariate pattern. Samples from the wet season exhibit greater variability and are more strongly associated with NAP and ANT concentrations.

Principal Component Analysis (PCA) is a widely applied multivariate approach in PAH source apportionment, as it reduces complex datasets into a few principal components that explain most of the variance and reflect groups of correlated compounds [68]. The biplot of PC 1 and PC 2 (Figure 5), which together accounted for 96.7% of the total variance (84.8% and 11.9%, respectively), revealed a separation between samples collected during the dry and wet seasons. Dry-season samples clustered tightly in the lower-left quadrant, reflecting uniform PAH compositions and lower mean concentrations. In contrast, samples from the wet season exhibited greater dispersion and were oriented along the NAP and ANT vector directions. Component loadings (Table S9) indicate that ANT had the strongest influence on PC1 (loading = 0.87794), followed by PHEN and NAP. Meanwhile, PC2 was primarily influenced by NAP (loading = 0.89129).

### 3.3. Correlation Patterns

Pearson correlation analysis between sampling sites supported the spatial and seasonal patterns observed. A positive correlation was found between sites U and M (r = 0.964), while moderate associations between U–O and M–O suggest similar contamination sources or hydrological connectivity among most sites. In contrast, weak or negative correlations with D indicate local variability, driven by distinct inputs or site-specific environmental processes (Supplementary Table S7). Inter-species correlation analysis also revealed strong relationships between individual PAHs. In particular, PHEN and ANT were strongly correlated (r = 0.824), while NAP showed moderate correlation with both compounds (Supplementary Table S8).

### 3.4. Results of Ecological Risk Assessment

HQ_water_ values were calculated for each site and sampling event using the maximum observed NAP concentrations. Table 3 summarizes the HQ_water_ values calculated for each site–season combination. All HQ_water_ values were below 0.01 (Table 1), ranging from 9.12 × 10^−5^ to 9.55 × 10^−3^, indicating insignificant ecological risk across the study area and seasonal sampling campaigns (Table 3). The highest HQ_water_ value (9.55 × 10^−3^), was observed at the upstream site during the September 2022 sampling campaign—corresponding to the wet season and the peak NAP concentrations. Although, this value approached the upper limit of the “insignificant” ecological risk category, it did not cross into the “low ecological risk” range.

Spatial patterns showed that HQ_water_ values were higher at upstream locations (U and M), particularly during wet seasons. In contrast, downstream sites (D and O), consistently exhibited lower HQ_water_ values. Seasonally, wet season events (September and November) show elevated HQ_water_ relative to dry season campaigns (March and February). However, none of the HQ_water_ values exceeded the threshold for “low risk”.

## 4. Discussion

### 4.1. Influence of Seasonal Hydrology on PAH Dynamics

The pronounced seasonal variation in Σ3PAHs concentrations observed in Caño La Malaria (mean = 745.79 ng/L during the wet season vs. 186.71 ng/L in the dry season) underscores the dominant role of rainfall-driven hydrological pulses in mobilizing PAHs within tropical urban wetlands. This enrichment during the wet period is consistent with findings from other tropical and subtropical freshwater systems [16,69,70], where elevated PAH concentrations were linked to runoff-driven inputs during rainy seasons. Across these systems, LMW-PAHs—particularly NAP, PHEN, and ANT—were consistently dominant, reflecting the compositional pattern found in our study.

Contrasting seasonal trends have been reported. For example, Na et al. [71] and Jiang et al. [72] observed higher PAH concentrations during the dry season in the East Liao River and coal-mining-impacted groundwater systems, attributing the lower wet-season levels to dilution and hydrodynamic flushing. Similar dry-season peaks were reported in the Olt River [73] and the Han River [74], suggesting that regional climate regimes, flow variability, and land-use characteristics can lead to divergent seasonal behaviors. These patterns suggest that regional climate, hydrology, and land use drive seasonal PAH dynamics, with the extensive impervious cover and limited riparian vegetation in Caño La Malaria amplifying runoff-driven transport of LMW-PAHs during intense tropical rainfall.

The predominance of LMW-PAHs—especially NAP—during the wet season in Caño La Malaria reflects a trend reported in other tropical aquatic systems, including the Subarnarekha Estuary [75], Gaoqiao wetland sediments [76], and the Sombreiro River Estuary [77]. Elevated temperatures during warmer months may further enhance the dissolution and water-phase partitioning of lighter PAHs [78], contributing to the seasonal increase in aqueous concentrations observed in our study. Dominance of LMW-PAHs has also been reported in highly industrialized watersheds, such as the Kolo Creek [79] and the Kuye River [80], reinforcing their utility as indicators of recent pyrogenic input and their susceptibility to hydrological mobilization. In Caño La Malaria, Σ3PAHs concentrations reached up to 2198.8 ng/L—exceeding values reported in many comparable tropical freshwater systems—highlighting the effects of sustained anthropogenic pressure, deficient stormwater infrastructure, and direct runoff inputs in this densely urbanized wetland catchment.

Compared to other global wetland systems, PAH levels in Caño La Malaria are elevated. In the Anzali Wetland, Iran, total PAH concentrations (Σ16PAHs) in surface waters ranged from 5.14 to 253.37 ng/L, with a mean of 78.31 ng/L, dominated by LMW congeners [81], values that overlap with our measured Σ3PAHs despite the narrower compound scope of this study. In the Hoor Al-Azim Wetland, Σ11PAHs ranged from 15.3 to 160.15 ng/L [82], while in the Shadegan Wetland, Σ16PAHs ranged from 42 to 136 ng/L with a mean of 78 ng/L, indicating low to slightly polluted conditions [83]. All of these reported values remain well below our recorded peak concentration of 2198.8 ng/L for Σ3PAHs in Caño La Malaria. These comparisons reveal significant human impact in Cucharillas Marsh and support the need for pollution control strategies.

In summary, seasonal hydrology plays a pivotal role in shaping PAH dynamics in Caño La Malaria, primarily by facilitating the mobilization and aqueous partitioning of LMW-PAHs during periods of intense rainfall. These findings not only corroborate patterns of rainfall-driven PAH mobilization observed in other tropical systems [32,84], but also contribute novel data from a Caribbean urban wetland. While variability across studies reflects differences in regional hydrology, emission profiles, and land use, the strong influence of wet-season runoff on the transport of pyrogenic and petrogenic PAHs emerges as a consistent feature of tropical and subtropical aquatic systems. It should be noted that these reported values correspond to Σ11–16PAHs, whereas our findings are limited to three LMW compounds (NAP, PHEN, ANT); thus, the comparison is illustrative of relative seasonal dynamics rather than magnitude-equivalent.

### 4.2. Spatial Distribution and Attenuation of PAHs

Although the Friedman test revealed no statistically significant differences in Σ3PAHs concentrations across the four sampling sites (*p* = 0.753). Elevated concentrations at U and M sites—particularly during high-rainfall events—suggest local inputs from adjacent roadways and potential illicit discharges, in addition to diffuse runoff [38,85]. In contrast, D and O sites consistently showed lower concentrations, likely due to hydrological dilution, sorption to suspended particulates, and gradual deposition along the canal’s flow path [86].

This U-to-D gradient aligns with patterns observed in other urban aquatic systems. For example, elevated PAHs have been reported in the Imiringi River due to vehicular emissions and localized combustion [87], and along the Nile River downstream of wastewater and industrial discharge zones [68]. Similar upstream enrichment has also been observed in the Lipu River [88], the Damodar River Basin [89], and the Guanzhong River in the Danjiangkou Reservoir, where fossil fuel combustion dominates [64]. These spatial trends are further corroborated by findings in Ho Chi Minh City and the East Liao River, where PAH concentrations were higher in densely urbanized, high-traffic areas [70,71]. A consistent pattern emerges in which upstream and highly urbanized segments function as hotspots of PAH contamination, whereas downstream reaches reflect the combined influences of hydrological dilution, sedimentation, and pollutant attenuation, particularly within aquatic coastal environments.

Beyond hydrological controls, land-use patterns exert influence on the spatial distribution of PAHs in Caño La Malaria. As demonstrated in the Sombreiro Estuary and Kuye River, where extreme PAH concentrations were detected near oil activity [77,80]. Although Σ3PAHs in Caño La Malaria were lower than in oil-impacted rivers, the system still exhibited spatial heterogeneity linked to anthropogenic pressure. This pattern parallels observations in Baiyangdian Lake, where coal and biomass combustion contributed to PAH variability modulated by both source proximity and hydrological retention [90]. Comparable trends in Dong and Tangxun Lakes, Wang Lake Wetland, and Brazilian estuarine systems further support that spatial PAH heterogeneity in urbanized tropical wetlands is shaped by a combination of land use and flow dynamics [91,92,93]. These studies indicate that the spatial heterogeneity observed in Caño La Malaria reflects broader patterns in which land-use intensity and hydrological processes modulate PAH distributions in urbanized wetland systems.

In summary, the spatial distribution of Σ3PAHs in Caño La Malaria reflects localized contamination at U and M sites, driven by road runoff, stormwater discharges, and surrounding land use. Although concentrations gradually decline toward D and O sites—suggesting attenuation through hydrological dilution, sedimentation, or sorption—elevated upstream levels, particularly during the wet season, highlight the persistent influence of urban inputs near the canal headwaters. These findings may inform the prioritization of upstream pollution controls and stormwater management strategies in similarly urbanized wetland systems.

### 4.3. Source Apportionment of PAHs

Compositional profiles of PAHs in Caño La Malaria revealed distinct seasonal trends. NAP was the most abundant compound during the dry season, while PHEN and ANT increased notably during the wet season. This is consistent with our observations, where NAP contributed up to 98% of Σ3PAHs in March 2022, while PHEN and ANT together accounted for more than 70% at several sites in September and November. These shifts reflect changes in transport mechanisms: volatilization and solubility-driven movement dominate in dry periods [94], whereas particle-bound inputs and surface runoff prevail during rainfall events [16]. Diagnostic ratios [ANT/(ANT + PHEN) > 0.10; PHEN/ANT < 10] consistently pointed to pyrogenic sources, implicating vehicular emissions and biomass combustion [95]. The correlation between PHEN and ANT (r = 0.824) reinforces this common origin and behavior under combustion-influenced regimes. In late-season samples (February 2023), PHEN overtook ANT as the predominant PAH at downstream sites, indicating shifting dominance even within the wet-to-dry seasonal transition. Seasonal compositional shifts observed in Caño La Malaria suggest the combined influence of transport mechanisms and combustion-derived sources.

PCA was applied to differentiate seasonal variability in PAH profiles and support source apportionment. PC1 was primarily influenced by ANT, followed by PHEN and NAP, together explaining 96.7% of the total variance. ANT and PHEN are associated with pyrogenic emissions from industrial and residential combustion processes [96], while NAP is often considered a general marker of petrogenic inputs such as oil spills and petroleum leaks [81,97]. Comparable PCA-based approaches in other riverine systems have similarly identified combustion-derived PAHs as major drivers, with additional contributions from petroleum-related inputs depending on local activities and hydrological settings [38,87]. In Caño La Malaria, the loadings of ANT and NAP suggest pyrogenic and petrogenic influences, in which the combined impact of combustion and petroleum sources influence PAH composition.

NAP dominated PC2 in our analysis, a pattern consistent with studies that linked strong NAP loadings to petroleum-derived inputs such as oil leaks and refined fuel residues [76,81]. Grmasha et al. [68] further emphasized that NAP is a major constituent of diesel fuels and gasoline, produced through incomplete combustion [98], and in their reported PCA, NAP appeared as a dominant component of PC1. The presence of NAP in PC2 indicates petroleum-derived sources, while the contributions of ANT and PHEN in PC1 reflect combustion markers, together accounting for the observed seasonal variations in PAHs. Overall, these findings demonstrate that PCA not only separates dry and wet season profiles but also highlights the dual influence of combustion and petroleum-related sources in shaping PAH dynamics in Caño La Malaria.

These diagnostic ratios, compositional profiles, and PCA-derived source attributions are consistent with broader reports from tropical and subtropical aquatic systems. In the Damodar River Basin and the East Liao River, 3- and 4-ring PAHs—including PHEN, fluoranthene, and pyrene—were associated with coal combustion and urban activity [71,89]. Similarly, PHEN and NAP were dominant in the Nile River and Ho Chi Minh City, reflecting urban runoff and motor vehicle emissions [70,99]. NAP was the most abundant PAH in the Danjiangkou Reservoir and the Taige Canal, attributed to petroleum combustion and diesel emissions, closely to the profiles observed in Caño La Malaria [64,100]. The compositional similarities between Caño La Malaria and other urban aquatic systems reveal that combustion-derived inputs are the dominant and persistent drivers of PAH behavior in this wetland.

Urban infrastructure facilitates the mobilization of combustion-derived PAHs, particularly in densely populated tropical catchments. Roads and stormwater systems promote the transfer of particle-bound compounds into aquatic systems, a dynamic also observed near PR-5 and PR-165 sampling sites in our study area [101]. This process is further amplified during the wet season, when increased runoff remobilizes PAHs previously adsorbed to soils and sediments [102]. Similar seasonal enrichment of LMW-PAHs—especially NAP—in urban watersheds suggest the influence of rainfall-driven inputs on PAH dynamics [103,104]. These patterns align with our findings in Caño La Malaria, where combustion-derived LMW-PAHs consistently dominated across both dry and wet seasons. The persistence of pyrogenic markers throughout the study period indicates chronic anthropogenic pressure from vehicular traffic, industrial activity, and stormwater discharge [38]. Altogether, the compositional and multivariate patterns observed in Caño La Malaria underscore the predominance of combustion-derived PAHs, with episodic petroleum inputs amplified by rainfall and stormwater infrastructure. These dynamics highlight the vulnerability of this tropical urban wetland to chronic anthropogenic pressure and establish the importance of source-specific management strategies.

### 4.4. Risk Implications and Management Recommendations

The concentrations of PAHs measured in Caño La Malaria frequently exceed established national and international environmental guidelines. The maximum concentrations for NAP (0.649 µg/L) remained below its benchmark of 1.1 µg/L, while PHEN (0.557 µg/L) and ANT (1.314 µg/L) notably exceeded their respective freshwater screening benchmarks (0.4 and 0.012 µg/L) [105]. Although widely used classification frameworks define ∑PAH contamination levels as slight (0–100 ng/L), mild (100–1000 ng/L), moderate (1000–5000 ng/L), and severe (>5000 ng/L) [106,107], these thresholds are based on the full suite of 16 priority PAHs and therefore cannot be directly applied to our dataset of three LMW PAHs. Nevertheless, the maximum concentrations observed here (up to 2198.8 ng/L) fall within the numerical range that in other systems corresponds to mild-to-moderate contamination, suggesting that our partial dataset may still reflect ecologically relevant pollution levels. When compared to chronic freshwater quality criteria, PHEN concentrations exceeded the 0.3 µg/L benchmark, reaching up to 0.561 µg/L, while NAP and ANT remained below their respective thresholds of 1 µg/L and 4 µg/L [108]. According to EU guidelines for inland surface waters, NAP concentrations did not exceed the threshold of 2000 ng/L, whereas ANT surpassed the 100 ng/L limit, particularly during the wet season [109,110]. Several wet season samples also exhibited ANT concentrations well above the 120 ng/L threshold set for marine organisms, with maximum values reaching 1342.05 ng/L [111]. These elevated concentrations indicate potential ecological risk under multiple regulatory frameworks, primarily reflecting chronic or sublethal protection thresholds established by international guidelines, and therefore do not necessarily imply acute toxicity under present conditions.

Beyond regulatory benchmarks, the ecological significance of the observed concentrations is further supported by thresholds derived from the target lipid model [112] and guidelines for human health protection [113]. Σ16PAHs concentrations in the Euphrates River ranged from 464 to 992 ng/L, with higher levels downstream of urban areas [68]. In Dong and Tangxun Lakes (China), Yao et al. [93] reported Σ16PAHs ranging from 42.9 to 434.7 ng/L. Σ3PAHs in Caño La Malaria exceeded 2000 ng/L, even though only three compounds were analyzed, compared to the full set of USEPA priority Σ16PAHs in other studies [68]. However, it should be noted that while the cited studies reported Σ16PAHs, our findings are based on three LMW compounds. Thus, the comparison is not magnitude-equivalent but rather illustrative of relative enrichment in dissolved-phase PAHs in tropical urban wetlands. Also, surface water concentrations often exceeded 1000 ng/L, likely due to the canal proximity to urban areas upstream [114,115], shallow depth [116], organic-rich sediments [117], and hydrological connectivity [118] to the San Juan Bay Estuary. Together, these comparisons reinforce that current environmental benchmarks may underestimate site-specific risks in tropical urban wetlands and highlight the need for more comprehensive water quality standards for PAHs.

Effective mitigation of PAH pollution in Caño La Malaria requires a watershed-scale management approach that integrates urban planning, regulatory oversight, and ecosystem-based solutions. In highly urbanized watersheds, persistent PAH contamination has been linked to vehicular emissions and stormwater runoff, which are also prominent sources in the Cucharillas Marsh area [70]. Nevertheless, despite improvements in water quality, legacy pollutants and diffuse PAH sources remain challenging to control without long-term strategies [88]. Among recommended strategies, nature-based solutions offer promising low-cost and sustainable alternatives [119]. Constructed wetlands have proven effective in attenuating PAHs through plant uptake, microbial degradation, and sedimentation [19]. Complementary stormwater management strategies, such as bio-swales [120] and infiltration basins [121], can further reduce runoff-borne PAHs before they enter the aquatic system. When implemented as small ponds or detention features along tributaries or upstream stormwater channels draining into Caño La Malaria, these interventions could intercept runoff during high-intensity rainfall events and significantly mitigate PAH inputs to the canal.

Findings from Caño La Malaria reveal that partial compliance with existing benchmarks does not guarantee ecological safety, because the observed concentrations reveal potential ecological risks that existing benchmarks may underestimate. While our study was limited to three parent LMW-PAHs, we note that traditional PAH assessments focusing solely on parent compounds may underestimate total toxicity in aquatic systems by excluding substituted derivatives, such as nitrated and oxygenated PAHs, which the literature shows can occur at higher concentrations and exhibit greater toxicity than their parent analogues [96]. Thus, expanding the analytical scope to include these compounds in future studies—together with applying updated classification systems for PAH contamination [107,122]—would provide a more comprehensive assessment of ecological risk. In parallel with improved monitoring, management interventions should not be limited to isolated hotspots [123,124]. Instead, efforts should also prioritize upstream pollution controls [125], the restoration of riparian buffers [126], and enhancements to stormwater infrastructure [127]. Ensuring long-term resilience in Cucharillas Marsh will require integrated monitoring and watershed-scale actions—rather than hotspot-only interventions—to mitigate chronic PAH pollution and protect ecological and hydrological functions.

### 4.5. Ecological Effects

The ecological risk associated with NAP in Caño La Malaria was found to be negligible across all sampling sites and seasons, with HQ_water_ values ranging from 9.1 × 10^−5^ to 9.5 × 10^−3^. These values fall below the 0.01 threshold for “insignificant risk” suggesting negligible acute ecological threat under current environmental conditions. Even the highest HQ_water_—recorded at the upstream site during the wet season (September 2022)—remained within the insignificant risk range. These findings are consistent with assessments conducted in subtropical systems, where PAH-related risks increased during wet-season runoff events due to pollutant mobilization and elevated concentrations [64]. Similarly, in coastal environments like tidal creeks, hydrological pulses during wet periods facilitated contaminant transport and led to higher ecological risk values [62].

Although HQ_water_ values in Caño La Malaria were consistently low, a clear spatial gradient was observed, with higher values upstream than downstream. This pattern aligns with findings from other river systems, where elevated upstream ecological risks are attributed to limited dilution capacity and closer proximity to pollutant sources [71]. Similarly, PAH-related risks in urbanized tidal creeks influenced by industrial and domestic discharges underscore the role of human activities in amplifying contaminant exposure [62]. Upstream sectors of urban wetlands serve as key vulnerability hotspots, where targeted ecological monitoring and management efforts are needed [128]. The elevated HQ_water_ values observed at the upstream site correspond to the most urbanized section of the watershed, located in the upper reach of Caño La Malaria, where dense residential development and major roadway infrastructure are present.

In the context of each study’s ERA framework, findings across wetlands and estuarine systems under strong urban and industrial influence indicate a consistent pattern. In Shadegan Wetland (Iran), the risk of PAHs in water was classified as moderate for several compounds, with benzo[a]anthracene (BaA) reaching high risk levels at most sites, while overall sediment risk was lower [83]. Similarly, in the Hoor Al-Azim wetland (Iran), the authors reported high risk for BaA in water but generally low to moderate risk for most other PAHs, while sediments indicated low biological risk [82]. In Anzali Wetland (Iran), sediments overall showed no harmful biological effects, although site-specific exceedances occurred for fluorene and pyrene, and several compounds, including NAP and ANT, were frequently above negligible-effect thresholds [81]. Consistent with our finding of insignificant acute risk for dissolved NAP, Edku Wetland (Egypt) showed no observed ecological hazard during spring [129]. By contrast, in estuarine systems, several PAHs, including PHEN, pyrene, and fluorene, frequently exceeded ecological risk thresholds, with clear seasonal variability [130]. More recently, studies demonstrated that, even when PAH water risk was negligible [129], sediment-bound PAHs can pose major ecological hazards in areas with dense urban infrastructure [131]. These comparisons indicate that while Caño La Malaria is not pristine, its present surface-water PAH levels imply insignificant ecological risk (HQ_water_ < 0.01) relative to those reported for other impacted wetlands; nonetheless, future risk assessments should integrate sediments and biota for a complete evaluation.

The ecological implications are particularly relevant when considering vulnerable benthic species such as the *Callinectes sapidus*, which serves as a key ecological indicator in Puerto Rico’s coastal wetlands. The negligible HQ_water_ values obtained in this study, calculated using toxicity thresholds derived from *Callinectes sapidus*, studies confirm a low acute ecological risk under current PAH concentrations [64]. However, evidence indicates that this species bioaccumulates PAHs in its tissues under long-term exposure conditions—particularly during wet seasons—with PHEN and ANT showing persistence that suggests slow metabolic degradation [132]. Other studies found no physiological or molecular effects after short-term exposure to PAH-contaminated sediments, highlighting the importance of exposure duration and compartment in ecological risk assessment [133]. Although *Callinectes sapidus* does not biomagnify PAHs across trophic levels, it is known to bioaccumulate these compounds from sediments, porewater, and diet in estuarine environments, showing its role in shaping PAH bioavailability within aquatic food webs [134]. Additionally, comparative assessments in rivers have shown that crustaceans and mollusks often exhibit higher ecological risk than fish, reinforcing their importance in monitoring frameworks [135]. These findings reaffirm the utility of *Callinectes sapidus* as a sentinel species and highlight the need to consider long-term exposure, seasonal variability, and sediment-phase contamination in future assessments.

Despite the insights gained from this study, several limitations must be acknowledged to contextualize the ERA more accurately. The risk assessment was restricted to dissolved NAP and focused solely on acute toxicity endpoints. It did not account for sediment-associated PAHs, high molecular weight congeners, or sublethal and chronic toxic effects—factors which have been shown to elevate ecological risk in similar wetland systems [91,136]. However, LMW-PAHs can induce oxidative stress and endocrine disruption even at concentrations below acute toxicity benchmarks, particularly under chronic exposure scenarios [137,138]. Similarly, in estuarine studies, PAHs such as fluoranthene, pyrene, and BaA were major contributors to total ecological risk, particularly when sediment-phase concentrations were considered [130]. These limitations underscore the need for integrative ERA approaches in Caño La Malaria. Exceedances of guideline values for PHEN and ANT suggest potential chronic concerns, while acute HQ_water_ results for NAP indicate insignificant risk—complementary rather than contradictory frameworks.

## 5. Conclusions

This study presents the first spatial–seasonal assessment of three LMW-PAHs (NAP, PHEN, and ANT) in surface waters of Caño La Malaria, the main hydrological conduit of the Cucharillas Marsh urban wetland (Cataño, Puerto Rico). Seasonal variability reflected rainfall-driven runoff and upstream urban inputs, while downstream attenuation was consistent with dilution and sediment–sorption processes. Compositional shifts were evident across campaigns: NAP dominated in March 2022, ANT prevailed during the peak wet season, and PHEN became most abundant at several downstream sites. Source apportionment analyses indicated a predominance of pyrogenic inputs—principally vehicular emissions and biomass combustion—with wet-season samples more strongly associated with NAP and ANT, with episodic contributions from petroleum-related sources. Correlation patterns revealed strong upstream connectivity but local variability at site D, while the strong association between PHEN and ANT suggests a common combustion-derived origin.

Ecological risk characterization showed negligible acute risk from NAP. However, PHEN and ANT frequently exceeded international benchmarks, underscoring the potential for chronic or sublethal effects not captured by the ERA, which was restricted to acute endpoints for NAP. Peak wet-season concentrations reached levels comparable to moderate pollution categories. NAP and PHEN regularly exceeded freshwater guideline values, while ANT occasionally neared guideline values for marine organisms. These suggest that current benchmark values may underestimate site-specific risks in Caño La Malaria. This highlights the need for comprehensive frameworks that integrate sediments, biota, and substituted PAHs to more accurately capture long-term ecological hazards.

From a management perspective, the results emphasize the necessity of integrated, watershed-scale interventions, including upstream pollution control, restoration of riparian buffers, and improvements to stormwater infrastructure, complemented by nature-based solutions. Future monitoring should include sediments and biota, the full suite of 16 USEPA priority PAHs and their derivatives, and chronic toxicity endpoints. Given that this study was limited to surface waters and three parent LMW-PAHs, a logical next step is to incorporate additional PAHs and seasonal sediment sampling. Collectively, these insights provide a transferable framework to strengthen the assessment and management of PAH pollution in tropical urban wetlands.

## Figures and Tables

**Table 1:** Ecological risk classification based on HQ_water_ values.

HQ_water_ Range	Ecological Risk Category
HQ_water_ < 0.01	Insignificant
0.01 ≤ HQ_water_ < 0.1	Low
0.1 ≤ HQ_water_ < 1.0	Moderate
HQ_water_ ≥ 1.0	High

**Table 2:** Mean concentrations (±standard deviation) and concentration ranges of NAP, PHEN, and ANT measured in surface water from four sites along Caño La Malaria, Puerto Rico, during four seasonal sampling campaigns conducted between March 2022 and February 2023.

Date	Site	NAP Mean (ng/L) ± SD	Range	PHEN Mean (ng/L) ± SD	Range	ANT Mean (ng/L) ± SD	Range	Σ3PAHs (ng/L)
March 2022	U	6.20 ± 0.00	ND–6.20	1.20 ± 0.00	ND–1.20	ND	ND	7.40
	M	30.13 ± 28.46	10.00–50.25	8.10 ± 1.91	6.75–9.45	ND	ND	38.23
	D	30.50 ± 26.38	11.85–49.15	1.25 ± 0.00	ND–1.25	0.50 ± 0.00	ND–0.50	32.25
	O	34.73 ± 32.99	11.40–58.05	8.70 ± 0.00	ND–8.70	ND	ND	43.43
September 2022	U	485.10 ± 231.93	321.10–649.10	135.87 ± 48.11	85.65–181.55	203.40 ± 0.00	114.50–292.30	824.37
	M	337.60 ± 32.39	308.60–372.55	364.72 ± 47.34	321.55–415.35	227.25 ± 109.39	149.90–304.60	929.57
	D	232.12 ± 29.02	203.80–261.80	240.47 ± 33.11	218.50–278.55	171.95 ± 116.67	100.75–306.60	644.54
	O	281.62 ± 67.63	230.50–358.30	297.17 ± 81.43	224.35–385.10	133.75 ± 36.98	108.90–176.25	712.54
November 2022	U	327.80 ± 183.92	197.75–457.85	557.43 ± 0.00	554.05–560.80	1313.60 ± 0.00	1285.15–1342.05	2198.83
	M	41.48 ± 38.36	14.35–68.60	183.30 ± 167.02	65.20–301.40	301.08 ± 383.29	30.05–572.10	525.86
	D	34.35 ± 0.99	33.65–35.05	5.00 ± 0.49	4.65–5.35	27.68 ± 3.22	25.40–29.95	67.03
	O	6.98 ± 4.56	3.75–10.20	37.13 ± 12.48	28.30–45.95	19.48 ± 2.02	18.05–20.90	63.59
February 2023	U	223.66 ± 185.24	43.00–399.15	183.49 ± 191.92	5.60–359.70	213.04 ± 213.39	2.25–412.20	620.19
	M	43.76 ± 1.29	41.75–45.75	46.04 ± 9.44	31.95–54.90	20.57 ± 17.34	2.45–39.10	110.37
	D	26.73 ± 20.12	<LOQ–52.80	165.11 ± 61.73	115.35–255.00	86.87 ± 15.61	72.20–112.95	278.71
	O	16.12 ± 11.70	3.60–28.80	228.98 ± 24.01	204.50–251.00	118.01 ± 4.33	112.30–123.50	363.11

Sites: upstream (U); midstream (M); downstream (D), and outlet to San Juan Bay (O). Values in the first column of each PAH represent mean ± standard deviation across replicates for each site and date. ND: Not detected; below limit of detection (LOD). <LOQ–xx: Range of replicates where at least one result was <LOQ and the maximum replicate yielded the stated value (xx). Σ3PAHs = sum of NAP, PHEN, and ANT concentrations. Range = minimum–maximum concentrations measured per site.

**Table 3:** Hazard Quotient Summary by Site and Sampling Date. PNEC value: 68,000 ng/L, based on acute toxicity data for *Callinectes sapidus* [67].

Date	Site	Max. NAP (ng/L)	HQ_water_ (ng/L)	Ecological Risk Classification
March 2022	U	6.20	9.12 × 10^−5^	Insignificant
	M	50.25	7.39 × 10^−4^	Insignificant
	D	49.15	7.23 × 10^−4^	Insignificant
	O	58.05	8.54 × 10^−4^	Insignificant
September 2022	U	649.10	9.55 × 10^−3^	Insignificant
	M	372.55	5.48 × 10^−3^	Insignificant
	D	261.80	3.85 × 10^−3^	Insignificant
	O	358.30	5.27 × 10^−3^	Insignificant
November 2022	U	457.85	6.73 × 10^−3^	Insignificant
	M	68.60	1.01 × 10^−3^	Insignificant
	D	35.05	5.15 × 10^−4^	Insignificant
	O	10.20	1.50 × 10^−4^	Insignificant
February 2023	U	399.15	5.87 × 10^−3^	Insignificant
	M	45.75	6.73 × 10^−4^	Insignificant
	D	52.80	7.76 × 10^−4^	Insignificant
	O	28.80	4.24 × 10^−4^	Insignificant

## Data Availability

The data presented in this study are contained within the article and are available from the corresponding author upon reasonable request.

## Supplementary Materials

The following supporting information can be downloaded at: https://www.mdpi.com/article/10.3390/toxics13100860/s1, Table S1. Physical properties and chemical structure of the 16 USEPA priority PAHs [138,139,140,141]; Table S2: Calibration curve parameters and calculated limits of detection (LOD) and quantification (LOQ) for naphthalene (NAP), phenanthrene (PHEN), and anthracene (ANT) in surface water samples; Table S3: Relative standard deviation (RSD%) and coefficient of variation (CV) for naphthalene (NAP), phenanthrene (PHEN), and anthracene (ANT) across all sites and sampling dates; Table S4: Shapiro–Wilk test for normality of Σ3PAHs concentrations; Table S5: Friedman test for seasonal differences in Σ3PAHs concentrations; Table S6: Friedman test for spatial differences in Σ3PAHs concentrations among sampling sites across all campaigns; Table S7: Pearson correlation matrix of Σ3PAHs concentrations between sampling sites in Caño La Malaria; Table S8: Pearson correlation matrix of naphthalene (NAP), phenanthrene (PHEN), and anthracene (ANT) concentrations in Caño La Malaria surface water; Table S9: Loadings of PAH compounds on the first three principal components derived from principal component analysis (PCA) of PAH concentrations in surface water samples from Caño La Malaria.

## Abbreviations

The following abbreviations are used in this manuscript:

PAHs	polycyclic aromatic hydrocarbons
LMW-PAHs	low molecular weight polycyclic aromatic hydrocarbons
HMW-PAHs	high molecular weight polycyclic aromatic hydrocarbons
NAP	naphthalene
PHEN	phenanthrene
ANT	anthracene
BaA	benzo[a]anthracene
Σ3PAHs	sum of three low molecular weight PAHs (naphthalene, phenanthrene, anthracene)
DCM	dichloromethane
PCA	Principal Component Analysis
USEPA	United States Environmental Protection Agency
GC-MS	Gas Chromatography–Mass Spectrometry
ng/L	Nanograms per Liter
U	upstream
M	midstream
D	downstream
O	outlet
RSD	relative Standard Deviation
EU	European Union
ND	nondetected
ERA	ecological risk assessment
HQ	hazard quotient
EC_water_	environmental concentration in water
PNEC_water_	predicted no-effect concentration in water
L(E)C_50_	lethal (or effect) concentration for 50% of organisms
AF	assessment factor
