# Supplementary material for: Occurrence and Distribution of Three Low Molecular Weight PAHs in Caño La Malaria, Cucharillas Marsh (Cataño, Puerto Rico): Spatial and Seasonal Variability, Sources, and Ecological Risk
Source: Toxics. 2025 Oct 11;13(10):860. doi: 10.3390/toxics13100860 (PMC12568084; doi:10.3390/toxics13100860)
Supplement: Supplementary file 1 [file toxics-13-00860-s001.zip › toxics-3883078-supplementary.pdf]

Supplementary Material

Occurrence and Distribution of Three Low Molecular Weight PAHs in Caño La Malaria, Cucharillas Marsh (Cataño, Puerto Rico): Spatial and Seasonal Variability, Sources, and Ecological Risk

Pedro J. Berríos-Rolón, Francisco Márquez* and María C. Cotto*

Nanomaterials Research Group, Department of Physics, Chemistry, and Mathematics, School of Natural Sciences and Technology, Universidad Ana G. Méndez-Gurabo Campus, Gurabo, PR 00778, USA; berriosp1@uagm.edu (P.J.B.-R); mcotto48@uagm.edu (M.C.C.); fmarquez@uagm.edu (F.M.)

* Correspondence: mcotto48@uagm.edu (M.C.C.); fmarquez@uagm.edu (F.M.); Tel.: +1-787-743-7979 (ext. 4491) (M.C.C.); +1-787-743-7979 (ext. 4250) (F.M.)

Contents

Contents…….. 1

Table S1. Physical properties and chemical structure of the 16 USEPA priority PAHs…… 2

Table S2. Calibration curve parameters and calculated limits of detection (LOD) and quantification (LOQ) for naphthalene (NAP), phenanthrene (PHEN), and anthracene (ANT) in surface water samples…… 3

Table S3. Relative standard deviation (RSD%) and coefficient of variation (CV) for naphthalene (NAP), phenanthrene (PHEN), and anthracene (ANT) in surface water samples across all sites and sampling dates…….. 4

Table S4. Shapiro-Wilk test for normality of ∑3PAHs concentrations…… 4

Table S5. Friedman test for seasonal differences in ∑3PAHs concentrations…….. 5

Table S6. Friedman test for spatial differences in ∑3PAHs concentrations among sampling points across all campaigns…….. 5

Table S7. Pearson correlation matrix of ∑3PAHs concentrations between sampling points in Caño La Malaria. ……5

Table S8. Pearson correlation matrix of Naphthalene (NAP), Phenanthrene (PHEN), and Anthracene (ANT) concentrations in Caño La Malaria surface water…… 6

Table S9. Loadings of PAH compounds on the first three principal components derived from principal component analysis (PCA) based on the correlation matrix of PAH concentrations in surface water samples from La Malaria Canal……6

Table S1. Physical properties and chemical structure of the 16 USEPA priority PAHs.

No.	Name	CS(a)	Rings	Class.(b)	MW(c)	BP(d)	MP(e)	S(f)	Log K_{ow}(g)	VP(h)
1	Naphthalene	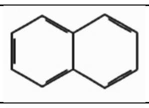	2	LMW	128.17	209	80	31.0	3.37	$8.89E^{-2}$
5	Phenanthrene	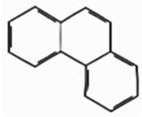	3	LMW	178.23	326	136	1.1	4.57	$6.80E^{-4}$
6	Anthracene	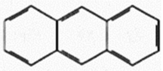	3	LMW	178.23	326	136	0.045	4.54	$2.55E^{-5}$

(a) CS: Chemical structure [1]; (b) Classification as LMW-PAHs and HMW-PAHs; (c) MW: Molecular weight in g/mol [1]; (d) BP: Boiling point in °C [2]; (e) MP: Melting point in °C [2]; (f) S: Water solubility at 25°C [3]; (g) Log K_{ow}: Octanol water partitioning [4]; (h) VP: Vapor pressure mmHg at 25°C [3].

Table S2. Calibration curve parameters and calculated limits of detection (LOD) and quantification (LOQ) for naphthalene (NAP), phenanthrene (PHEN), and anthracene (ANT) in surface water samples.

PAH	Sampling Date	m	b	SE	R^2	LOD (ng/L)	LOQ (ng/L)
NAP	March 20, 2022	17222.00	4497.60	96.47	0.99	0.02	0.06
PHEN	March 20, 2022	15921.54	21531.43	124.55	0.99	0.03	0.08
ANT	March 20, 2022	16748.28	23566.25	155.62	0.99	0.03	0.09
NAP	September 5, 2022	35041.86	-573995.46	3962.82	0.96	0.37	1.13
PHEN	September 5, 2022	36392.28	-342784.32	2208.12	0.99	0.20	0.61
ANT	September 5, 2022	32534.77	-312128.99	2507.32	0.98	0.25	0.77
NAP	November 6, 2022	10672.11	13989.38	125.28	0.99	0.04	0.12
PHEN	November 6, 2022	8807.07	7944.81	106.78	0.99	0.04	0.12
ANT	November 6, 2022	7422.32	1646.15	68.23	0.99	0.03	0.09
NAP	February 26, 2023	11827.49	-69372.44	1881.82	0.88	0.53	1.59
PHEN	February 26, 2023	11738.98	-84735.15	2107.36	0.86	0.59	1.80
ANT	February 26, 2023	7794.64	-49853.07	1220.31	0.89	0.52	1.57

m = slope of the calibration curve; b = y-intercept; R^2 = coefficient of determination; SE = standard error of the y-intercept; LOD = limit of detection; LOQ = limit of quantification. LOD and LOQ were calculated using the formulae LOD = 3.3 × SE / m and LOQ = 10 × SE / m.

Table S3. Relative standard deviation (RSD%) and coefficient of variation (CV) for naphthalene (NAP), phenanthrene (PHEN), and anthracene (ANT) in surface water samples across all sites and sampling dates.

		NAP		PHEN		ANT	
**Date**	**Site**	**RSD (%)**	**CV**	**RSD (%)**	**CV**	**RSD (%)**	**CV**
March 20, 2022	U	0.00	0.00	0.00	0.00	0.00	0.00
	M	94.48	0.94	23.57	0.24	0.00	0.00
	D	86.48	0.86	0.00	0.00	0.00	0.00
	O	94.99	0.95	0.00	0.00	0.00	0.00
September 5, 2022	U	47.81	0.48	35.41	0.35	0.00	0.00
	M	9.59	0.10	12.98	0.13	48.14	0.48
	D	12.50	0.13	13.77	0.14	67.85	0.68
	O	24.01	0.24	27.40	0.27	27.65	0.28
November 6, 2022	U	56.11	0.56	0.00	0.00	0.00	0.00
	M	92.49	0.92	91.12	0.91	127.31	1.27
	D	9.90	0.10	2.88	0.03	11.63	0.12
	O	65.39	0.65	33.62	0.34	10.35	0.10
February 26, 2023	U	82.82	0.83	104.59	1.05	0.00	0.00
	M	2.95	0.03	20.51	0.21	84.29	0.84
	D	75.27	0.75	37.39	0.37	17.97	0.18
	O	72.61	0.73	10.49	0.10	3.67	0.04

U = Upstream; M = Midstream; D = Downstream; O = Outlet; RSD = Relative standard deviation; CV = Coefficient of variation.

Table S4. Shapiro-Wilk test for normality of ∑3PAHs concentrations.

**Variable**	**n**	**SW statistic**	**SW critical value (α = 0.05)**	**p-value**	**Normality conclusion**
Σ 3PAHs (ng/L)	16	0.000	0.887	p<0.01	Not normally distributed

Note: According to the Shapiro-Wilk test, the ∑3PAHs concentration data do not follow a normal distribution (p < 0.01), justifying the use of non-parametric statistical methods in subsequent analyses.

Table S5. Friedman test for seasonal differences in ∑3PAHs concentrations.

**Comparison Type**	**Test statistic (χ^2)**	Critical value (α = 0.05)	p-value	Significance
Seasonal variability (dry vs. wet)	21	3.84	4.59×10^{-6}	Significant (reject H_0)

Note: The Friedman test revealed statistically significant seasonal differences in ∑3PAHs concentrations across the four sampling campaigns (March, September, November 2022 and February 2023). The null hypothesis of equal medians across seasons was rejected (p < 0.001), indicating that PAH concentrations were influenced by seasonal hydrological conditions.

Table S6. Friedman test for spatial differences in ∑3PAHs concentrations among sampling points across all campaigns.

**Comparison Type**	**Blocks**	**Treatments**	**Test statistic (χ^2)**	**Critical value (α = 0.05)**	**p-value**	**Conclusion**
Sampling points over time	4	4	1.20	7.81	0.753	Nosignificant difference (fail to reject H_0)

Note: The Friedman test was applied to compare ∑3PAHs concentrations among the four sampling points across all campaigns. The analysis showed no statistically significant spatial differences (p = 0.753), indicating relatively uniform contamination levels among sites during the study period.

Table S7. Pearson correlation matrix of ∑3PAHs concentrations between sampling points in Caño La Malaria.

**Sampling Sites**	**U**	**M**	**D**	**O**
U	1.000	0.964	-0.085	0.550
M		1.000	-0.175	0.571
D			1.000	0.425
O				1.000

Note: The matrix presents Pearson correlation coefficients (r) of ∑3PAHs concentrations between sampling sites (P1–P4). Strong positive correlations between P1 and P2 (r = 0.964), and moderate correlations with P4, suggest possible shared contamination sources. Negative or weak correlations with P3 may reflect distinct hydrological or contamination patterns.

Table S8. Pearson correlation matrix of Naphthalene (NAP), Phenanthrene (PHEN), and Anthracene (ANT) concentrations in Caño La Malaria surface water.

**PAH Species**	NAP	PHEN	ANT
NAP	1.000	0.650	0.499
PHEN		1.000	0.824
ANT			1.000

Note: Pearson correlation analysis revealed strong positive associations between phenanthrene and anthracene (r = 0.824), and moderate correlations between naphthalene and the other two PAHs. These relationships suggest common pyrogenic sources and similar environmental behavior among the compounds.

Table S9. Loadings of PAH compounds on the first three principal components derived from principal component analysis (PCA) based on the correlation matrix of PAH concentrations in surface water samples from La Malaria Canal.

**Variable (PAH)**	PC1	PC2	PC3
NAP	0.27113	0.89129	-0.36343
PHEN	0.3946	0.24147	0.88656
ANT	0.87794	-0.38378	-0.28623

Note: Loadings represent the correlation of each compound with the principal component axes. PCA was performed using a correlation matrix.

References

1. National Center for Biotechnology Information PubChem Dataset 2023.
2. Shen, H. *Polycyclic Aromatic Hydrocarbons*; Springer Theses; Springer Berlin Heidelberg: Berlin, Heidelberg, 2016; ISBN 978-3-662-49678-7.
3. Agency for Toxic Substances and Disease Registry Toxicological Profile for Polycyclic Aromatic Hydrocarbons 1996.
4. Mackay, D.; Shiu, W.-Y.; Shiu, W.-Y.; Lee, S.C. *Handbook of Physical-Chemical Properties and Environmental Fate for Organic Chemicals*; 0 ed.; CRC Press, 2006; ISBN 978-0-429-15007-4.
